# LITHOPHONE: Improving lncRNA Methylation Site Prediction Using an Ensemble Predictor

**DOI:** 10.3389/fgene.2020.00545

**Published:** 2020-06-09

**Authors:** Lian Liu, Xiujuan Lei, Zengqiang Fang, Yujiao Tang, Jia Meng, Zhen Wei

**Affiliations:** ^1^School of Computer Sciences, Shannxi Normal University, Xi'an, China; ^2^Department of Biological Sciences, Xi'an Jiaotong-Liverpool University, Suzhou, China

**Keywords:** m^6^A, lncRNA, site prediction, epitranscriptome, ensemble model

## Abstract

*N*^6^-methyladenosine (m^6^A) is one of the most widely studied epigenetic modifications, which plays an important role in many biological processes, such as splicing, RNA localization, and degradation. Studies have shown that m^6^A on lncRNA has important functions, including regulating the expression and functions of lncRNA, regulating the synthesis of pre-mRNA, promoting the proliferation of cancer cells, and affecting cell differentiation and many others. Although a number of methods have been proposed to predict m^6^A RNA methylation sites, most of these methods aimed at general m^6^A sites prediction without noticing the uniqueness of the lncRNA methylation prediction problem. Since many lncRNAs do not have a polyA tail and cannot be captured in the polyA selection step of the most widely adopted RNA-seq library preparation protocol, lncRNA methylation sites cannot be effectively captured and are thus likely to be significantly underrepresented in existing experimental data affecting the accuracy of existing predictors. In this paper, we propose a new computational framework, **LITHOPHONE**, which stands for **l**ong noncod**i**ng RNA me**th**ylati**o**n sites **p**rediction from sequence c**h**aracteristics and gen**o**mic i**n**formation with an **e**nsemble predictor. We show that the methylation sites of lncRNA and mRNA have different patterns exhibited in the extracted features and should be differently handled when making predictions. Due to the used experiment protocols, the number of known lncRNA m^6^A sites is limited, and insufficient to train a reliable predictor; thus, the performance can be improved by combining both lncRNA and mRNA data using an ensemble predictor. We show that the newly developed LITHOPHONE approach achieved a reasonably good performance when tested on independent datasets (AUC: 0.966 and 0.835 under full transcript and mature mRNA modes, respectively), marking a substantial improvement compared with existing methods. Additionally, LITHOPHONE was applied to scan the entire human lncRNAome for all possible lncRNA m^6^A sites, and the results are freely accessible at: http://180.208.58.19/lith/.

## Introduction

RNA modifications include more than 150 different types, among which *N*^6^-methyladenosine (m^6^A) has attracted the most attention due to its universality and various biological functions (Fu et al., 2014; Liu and Jia, 2014; Meyer and Jaffrey, 2014). The m^6^A RNA methylation denotes that the amino group on the sixth carbon atom of adenine is modified by a methyl group, usually occurring in the conservative sequence RRACH (R = G, A; H = A, C, or U) or GGAC (Dominissini et al., 2012). The universality of m^6^A is reflected in the following two aspects. On the one hand, it appears in almost all RNA transcripts, including coding and non-coding ones (Dominissini et al., 2012; Alarcón et al., 2015b). On the other hand, it is enriched near the stop codon, 3′ untranslated regions, and the last exon region of mRNA (Liu et al., 2014, 2015). Recent studies (Alarcón et al., 2015a; Roost et al., 2015) showed that as a common molecular tag, m^6^A modification is involved in many important biological processes, including RNA localization and degradation (Wang et al., 2014), RNA structural dynamics (Roost et al., 2015; Song et al., 2020), variable splicing (Wang et al., 2014), primary microRNA process (Chen et al., 2015a; Geula et al., 2015), cell differentiation and adaptation, and circadian clock regulation (Fustin et al., 2013). It is also associated with protein translation, obesity, abnormal brain development, and a few other diseases (Peng et al., 2016).

Long non-coding RNA (lncRNA) refers to a class of RNAs that have no coding potentials and are of a length >200 nucleotides (nt). Studies have shown that lncRNA plays an important role in many life activities, such as dosage compensation effect, epigenetic regulation, cell cycle regulation, and cell differentiation regulation (Qureshi et al., 2010; Peng et al., 2016). Recent epitranscriptome analysis has shown that thousands of lncRNAs contain a large number of methylation sites (Shafik et al., 2016). For example, m^6^A methylation is important for the silencing or inactivation of the X chromosome gene mediated by lncRNA XIST (Patil et al., 2016). The m^6^A methylation of XIST is completed by recruiting the complex composed of RBM15 (RNA-binding motif protein 15)/RBM15B-WTAP-METTL3 to the specific region of XIST, the methylation recognition protein (reader) YTHDC1 then binds to this region and recruits silencing proteins to complete the whole gene suppression process. Moreover, the m^6^A methylation of MALAT1 regulates pre-RNA synthesis. It was found that MALAT1 could carry this methylation in the stem ring structure. After m^6^A methylation, the binding ability of the gene to the hnRNP C protein was enhanced (Nian et al., 2015). In addition, m^6^A methylation can regulate lncRNA FOXM1-AS to promote the proliferation of cancer cells (Zhang et al., 2017; Song et al., 2020), and regulate lncRNA1281 to affect the differentiation of mouse embryonic stem cells (Yang et al., 2018).

With the development of high-throughput sequencing (HTS) technology, a new field of epitranscriptome analysis has emerged. The invention of MeRIP-Seq in 2012 (Meyer et al., 2012) presented the first technique to detect the m^6^A spectrum in the whole transcriptome, during which RNA was randomly fragmented into short pieces of around 100 nt long; the fragments containing methylation modification were captured using the specific antibodies, and then subjected to sequencing to generate the IP samples; meanwhile, an input control sample was generated in parallel to serve as the background. Tools like MACS (Zhang et al., 2008), exomePeak (Meng et al., 2013), or other peak calling methods are usually used to detect m^6^A peaks with a length of about 100 nt (Chen et al., 2017). It is possible to further narrow down the precise location of m^6^A sites by searching for the m^6^A conforming DRACH motif in the detected peaks. However, since these methods cannot distinguish the random DRACH motifs from the real m^6^A-containing motifs nearby, a large number of false-positive m^6^A methylation sites is reported by MeT-DB (Liu et al., 2018) and RMBase (Xuan et al., 2018), as previously reported (Zhang et al., 2019). In addition to MeRIP-Seq, technologies with a single base resolution such as miCLIP (Bastian et al., 2015) and m^6^A-CLIP (Shengdong et al., 2015) have been developed. However, due to the high difficulty and cost of base-resolution experiments, these technologies have not been widely used compared with MeRIP-Seq.

*In silico* methods to predict methylation sites based on machine learning (ML) approaches have been increasingly popular in recent years. For example, Chen et al. proposed the first ML method to predict RNA methylation sites in 2015, called “iRNA-Methyl” (Chen et al., 2015b). This method used dinucleotide composition and physicochemical characteristics to construct the PseDNC in order to represent RNA sequences and used these as an input to support vector machines (SVMs) to predict the m^6^A methylation sites of *Saccharomyces cerevisiae*. Later, Zhou et al. (2016) used a variety of features to represent the sequence information, including the features of sequence coding, K-nearest base pair similarity and base pair frequency, to train the predictive model with the random forest (RF) method for the m^6^A methylation sites prediction in mammalians. MethyRNA (Chen et al., 2016) encoded RNA sequences using the nucleotides' chemical properties and their accumulated frequency information, and used SVM classifier to predict the methylation modification sites of *S. cerevisiae*. M6AMRFS (Qiang et al., 2018) represented the sequence features with dinucleotide binary encoding (DBE) and local position-specific dinucleotide frequency (LPDF), and predicted the methylation modification sites of *S. cerevisiae* m^6^A based on an eXtreme Gradient Boosting (XGBoost) classifier. Besides, a number of methods used deep learning (DL) approaches to predict m^6^A methylation sites. BERMP (Yu Huang et al., 2018) used the base coding and the frequency of each base in a sliding window of a certain length as the characteristics of the sequence information. Using trained Gated Recurrent Unit (GRU) classifier and RF classifier, the final prediction results are obtained by logical regression. In DeepM6ASeq (Zhang and Hamada, 2018), the sequence was encoded using a one-hot encoding scheme, and the methylation modification sites were then predicted using a deep learning model consisting of a convolutional neural network (CNN) layer and one bidirectional long short-term memory (BLSTM) layer. Gene2vec (Quan Zou et al., 2018) took the methylation status near the methylation site, a one-hot encoding, the RNA word embedding feature, and the context word embedding feature as sequence features, used them respectively as an input to a CNN, and used a devoting method to predict the location. Deep-m6A (Zhang Sy et al., 2019) took the product of a one-hot encoding of the sequence characteristics and the sites' reads count in the IP samples as an input to predict m^6^A sites using a CNN. In addition, PRNAm-PC (Liu et al., 2016), RAM-ESVM (Wei et al., 2017a), AthMethPre (Xiang et al., 2016), and other methods (Chen et al., 2015c; Li et al., 2016; Zhao et al., 2018; Liu et al., 2020) can also be used to predict m^6^A methylation sites. Although all these methods can predict RNA methylation sites, they are entirely based on the sequence context information. Even when secondary structures or other advanced features are used, the information is still directly extracted from the sequence without considering other potential and useful genomic features, referring to genome-related features that are not directly derived from sequences, including the secondary structure, gene annotation, transcription type, conservation, and many more. Recently, the method of WHISTLE (Zhang et al., 2019) combined sequence and genomic features to predict m^6^A sites and constructed the entire m^6^A epitranscriptome, showing that genomic features can also be very effective in the prediction of these sites and should be considered in the prediction framework.

Although the aforementioned methods can all perform general RNA methylation sites prediction, none of them was specifically considered or optimized for lncRNA methylation sites detection. Most of the currently existing experimental data use polyA selection when constructing the RNA-seq library; thus, lncRNAs will not be effectively captured since many of them are non-polyadenylated, and many lncRNA methylation sites are likely to be missed in the data generated from such protocol that would mainly contain the methylation sites information of mRNAs. As a result, the performance of site predictors trained with such data is likely to be limited when they are applied for the lncRNA methylation sites prediction task. The interplay between lncRNA and RNA methylation is now of an increasing interest to the science community and it is needed to develop a lncRNA-specific methylation sites prediction tool.

In this paper, we propose a new computational framework, **LITHOPHONE**, which stands for **l**ong noncod**i**ng RNA me**th**ylati**o**n sites **p**rediction from sequence c**h**aracteristics and gen**o**mic i**n**formation with an **e**nsemble predictor. LITHOPHONE uses a RF classifier to predict m^6^A methylation sites by extracting the physicochemical and frequency accumulation characteristics of the bases based on sequence information and multiple genomic features, and identify lncRNA methylation sites by combining the information from mRNA and lncRNA sites using an ensemble predictor.

## Materials and Methods

### Dataset Construction

For predicting the m^6^A methylation sites in lncRNA, we employed the ground truth data that was used in the WHISTLE project (Zhang et al., 2019), including six single-base resolution m^6^A experiments from six datasets obtained from five cell types (see Table 1): HEK293T, MOLM13, A549, CD8T, and HeLa, respectively, where HEK293T has two samples. The annotation information of lncRNA was obtained through Bioconductor via the TxDb.Hsapiens.UCSC.hg19.lincRNAsTranscripts R package. The positive m^6^A sites were defined as under the DRACH consensus motifs in at least two of the six datasets. The negative m^6^A sites were randomly selected from the non-positive DRACH adenosines on the full transcripts containing the positive sites. There were equal numbers of negative and positive sites for each set of the training data, and the underlying motifs were restricted on DRACH. In addition, no sites were reported from the regions that can be mapped to multiple genes.

**Table 1:** Single-base resolution m^6^A datasets in lncRNA m^6^A prediction.

**Cell**	**Note**	**References**
HEK293T	Abacm antibody	Bastian et al., 2015
HEK293T	Sysy antibody	Bastian et al., 2015
MOLM13		Vu et al., 2017
A549		Shengdong et al., 2015
CD8T		Shengdong et al., 2015
HeLa		Ke et al., 2017

Finally, 2,582 full transcript m^6^A sites in lncRNA were collected, including 1,291 positive sites and 1,291 negative ones, while 2,214 m^6^A sites were obtained in mature lncRNA mode with 1,107 positive sites and 1,107 negative ones. Four-fifths of the sites were randomly selected for training, and the rest was retained for testing under both full transcript and mature RNA modes, respectively. For comparison purposes, we also generated the matched data for mRNAs, including 57,105 positive sites and the same number of negative ones for the full transcript mode, and 54,476 positive sites and 54,476 negative ones for the mature RNA mode, respectively. There were many more mRNA methylation sites compared with the lncRNA sites, suggesting that the mRNA methylation sites usually dominate the epitranscriptome profiling results.

### Feature Representation

In this work, the sequence and genomic features were simultaneously used to represent a m^6^A site.

#### Sequence Features

A nucleotide in a 21-nt sequence around the DRACH motif was represented by a four-dimensional vector following the method of MethyRNA (Chen et al., 2016). Firstly, each kind of nucleotide in RNA, including adenine (A), guanine (G), cytosine (C), and uracil (U), was represented by three characteristics according to its different chemical characteristics. For example, there is only one ring structure in cytosine and uracil, while adenine and guanine have two rings; adenine and cytosine both contain an amino group, while guanine and uracil both contain a keto group; hydrogen bonds are strong in guanine and cytosine when forming the secondary structure, while they are weak in adenine and uracil. According to these three features, a three-dimensional vector *S* = (*x*_*i*_, *y*_*i*_, *z*_*i*_) could be used to represent a nucleotide:

(1) $$\begin{array}{l} x=\{\begin{array}{ccc} 1 & if & s\in \left\{A,G\right\}\\ 0 & if & s\in \left\{C,U\right\} \end{array},y=\{\begin{array}{ccc} 1 & if & s\in \left\{A,C\right\}\\ 0 & if & s\in \left\{G,U\right\} \end{array},z=\{\begin{array}{ccc} 1 & if & s\in \left\{A,U\right\}\\ 0 & if & s\in \left\{C,G\right\} \end{array} \end{array}$$

Therefore, based on the above-defined rules, the vectors (1,1,1), (0,1,0), (1,0,0), and (0,0,1) can be used to encode A, C, G, and U, respectively. Next, the base accumulation frequency was also considered to describe the distribution of each base in the sequence. This frequency was defined as the frequency of the *i*th base in the previous *i* bases. The density *f*_*i*_ of the *i*th base is calculated by *f*_*i*_ = *d*_*i*_*/i*, where *f*_*i*_ is the frequency of the occurrence of the *i*th base before *i* position density, and *d*_*i*_ is defined as the sum of the occurrences of the *i*th base in the previous *i* bases. For a sequence like “ACCUGAAUUG,” A occurs three times at the 1st, 5th, and 6th positions, so the cumulative frequencies are 1/1, 2/5, and 3/6, respectively. However, the cumulative frequencies of *C* are 1/2 and 2/3; those of *U* are 1/4, 2/8, and 3/9; and those of *G* are 1/5 and 2/10. According to the above-described chemical characteristics and frequency cumulative distribution characteristics, each base can be encoded using a four-dimensional vector.

#### Genomic Features

Sequence features can only reflect the characteristics of each base in the sequence, but they cannot represent the topological information of the RNA methylation sites; thus, 60 additional genomic features were generated to reflect this information for the RNA methylation prediction in lncRNA. These features are detailed as follows: genomic features 1–10 are the dummy variable features, which indicate whether the site is overlapped with the topological region on the major RNA transcript. In order to extract genomic features, the longest transcripts were selected to prevent the influence of transcription isoforms. All features were extracted using the transcriptional annotations of the hg19 TxDb package (Xuan et al., 2018). Genomic features 11–12 stand for the distances toward the splicing junctions. Features 13–14 represent the length of the transcript region containing the methylation site. Features 15–32 indicate the consistence motif to which the RNA methylation site belongs. Features 33–36 represent clustering indicators or motif clustering, which reflect the clustering effect of the RNA methylation sites. Features 37–40 are the scores related to the evolutionary conservation, including two Phast-Cons scores and two fitness consequences scores. Features 41–42 obtain the secondary structure information of the RNA using RNAfold (Gruber et al., 2015). RNA annotations related to m^6^A biology are features 43–55. Feature 56 is a dummy variable indicating whether the lncRNA is a miRNA target. Finally, features 57–60 include two *z*-scores of the isoform and exon number, and two *z*-scores of the GC content. Table S1 contains the detailed information of the genomic features considered in the prediction.

### Evaluation Metrics

In order to measure the prediction effect of the model, we used the measurements of sensitivity (Sn), specificity (Sp), accuracy (ACC), and Matthews correlation coefficient (MCC) to show the results of the model. The four indicators are respectively defined as follows:

(2) $$\begin{array}{l} S_{n}=\frac{TP}{TP+FN} \end{array}$$

(3) $$\begin{array}{l} S_{p}=\frac{TN}{TN+FP} \end{array}$$

(4) $$\begin{array}{l} ACC=\frac{TP+TN}{TN+FP+TP+FN} \end{array}$$

(5) $$\begin{array}{l} MCC=\frac{TP\times TN-FP\times FN}{\sqrt{\left(TP+FP\right)\left(TN+FN\right)\left(TP+FN\right)\left(TN+FP\right)}} \end{array}$$

where TP, TN, FP, and FN are the true positive, true negative, false positive, and false negative values, respectively. The sensitivity reflects the success rate of the positive sample prediction, and the specificity reflects the success rate of the negative sample prediction. A good prediction system should have both a high sensitivity and a high specificity at the same time. If the sensitivity is very high and the specificity is low, the false positive will be very high, while if the specificity is very high and the sensitivity is low, the false negative will be very high. Therefore, the forecasting system needs to comprehensively consider these two indicators. Matthews correlation coefficient is a comprehensive performance evaluation index considering unbalanced datasets. In addition, we plotted the receiver operating characteristic (ROC) curves and calculated the areas under the curves (as called “AUC”) to evaluate the prediction performance.

## Results and Discussion

### Comparing RF and Other Algorithm Performance Through Cross-Validation

In order to compare the prediction results of different algorithms, five different classifiers were used: RF (Liu, 2017; Wei et al., 2017b), SVM (Song et al., 2018), K-nearest neighbor (KNN) (Jia et al., 2016), logistic regression (LR) (Cha et al., 2015) and XGBoost (Chen and Guestrin, 2016). RF is a popular ML algorithm used to predict m^6^A RNA methylation, which was applied in SRAMP (Zhou et al., 2016) to predict mammalian m^6^A sites. SVM is another ML algorithm applied in computational biology, based on which the methods of MethyRNA (Chen et al., 2016) and RAM-ESVM (Wei et al., 2017a) were developed to predict RNA methylation sites. KNN is one of the most powerful methods in the data mining classification technology, and LR is an ML method with a simple algorithm and a high performance. XGBoost is frequently used in competitions and industry, and can be effectively applied to the tasks of classification, regression, and ranking; it was used in M6AMRFS (Qiang et al., 2018) to predict m^6^A sites in multiple species based on the sequence features. All methods were implemented using the corresponding R packages (see Table S2). In order to compare their performance, a 10-fold cross-validation was employed on the training datasets under the full transcript and mature lncRNA modes. The performance of the different classifiers is summarized in Table 2, which shows that RF achieved the best performance both under the full transcript mode and mature lncRNA mode with an AUC of 0.971 and 0.827, respectively.

**Table 2:** Performance under 10-fold cross-validation.

**Mode**	**Method**	**Evaluation metrics**				
		**Sn**	**Sp**	**ACC**	**MCC**	**AUC**
Full transcript	RF	0.923	0.938	0.930	0.861	0.971
	SVM	0.884	0.942	0.913	0.828	0.964
	KNN	0.5	0.501	0.500	0.001	0.945
	LR	0.881	0.944	0.912	0.827	0.962
	XGBoost	0.907	0.940	0.924	0.848	0.955
Mature lncRNA	RF	0.784	0.724	0.754	0.511	0.827
	SVM	0.738	0.713	0.725	0.451	0.796
	KNN	0.499	0.501	0.500	0.001	0.727
	LR	0.602	0.807	0.704	0.418	0.789
	XGBoost	0.645	0.697	0.671	0.345	0.722

### Independent Tests Suggest That lncRNA and mRNA Methylation Sites Possess Different Characteristics

Next, we independently tested the m^6^A sites on lncRNA in the full transcript and mature lncRNA modes. It is worth mentioning that none of the existing sites prediction methods differentiated between lncRNA and mRNA sites. Since mRNA sites are significantly over-represented in the data, it should dominate the performance assessment results. In the following tests, the mRNA and lncRNA sites were explicitly separated in both training and testing phases. Specifically, we used m^6^A sites from both mRNA and lncRNA for the training, and then as testing sites from the two categories as well. We used the training data in lncRNA to train in the full transcript mode, tested with the testing data of lncRNA and mRNA separately, then trained with the training data in mRNA and finally tested with the testing data of lncRNA and mRNA separately. The same method was used in the mature lncRNA mode. As shown in Table 3, the best performance was achieved when the training and testing data were matched, suggesting that lncRNA and mRNA methylation sites exhibited different characteristics. When using lncRNA data as training samples to predict m^6^A sites in lncRNA, the prediction performance (AUC = 0.966 and AUC = 0.821, under full transcript and mature RNA modes, respectively) was better than when we used mRNA data as training samples to predict the sites of lncRNA (AUC = 0.936 and AUC = 0.807, under full transcript and mature RNA modes, respectively). Similarly, this situation also occurs in predicting the sites of mRNA. When mRNA sites were used for training, the results achieved for testing the sites of mRNA were better than those of lncRNA. In addition, it can be seen that the method of RF can achieve the best prediction results in both cross-validation and independent testing among the five different prediction methods. Therefore, RF is chosen as a classifier to predict the methylation sites in lncRNA.

**Table 3:** Performance under independent test.

**Mode**	**Training data**	**Testing data**	**Method**	**Evaluation metrics**				
				**Sn**	**Sp**	**ACC**	**MCC**	**AUC**
Full transcript	lncRNA	lncRNA	RF	0.922	0.930	0.926	0.853	0.966
			SVM	0.903	0.934	0.919	0.838	0.963
			KNN	0.500	0.500	0.500	0.000	0.942
			LR	0.895	0.926	0.911	0.822	0.959
			XGBoost	0.922	0.903	0.913	0.826	0.947
	lncRNA	mRNA	RF	0.981	0.046	0.514	0.077	0.759
			SVM	0.984	0.051	0.518	0.098	0.678
			KNN	0.499	0.501	0.500	0.000	0.572
			LR	0.954	0.171	0.562	0.200	0.716
			XGBoost	0.908	0.250	0.579	0.209	0.697
	mRNA	lncRNA	RF	0.752	0.934	0.843	0.698	0.936
			SVM	0.744	0.899	0.822	0.651	0.905
			KNN	0.492	0.508	0.500	0.000	0.703
			LR	0.539	0.953	0.746	0.541	0.872
			XGBoost	0.721	0.891	0.806	0.622	0.869
	mRNA	mRNA	RF	0.846	0.833	0.839	0.679	0.913
			SVM	0.829	0.839	0.834	0.669	0.908
			KNN	0.499	0.501	0.500	0.001	0.798
			LR	0.717	0.896	0.806	0.623	0.898
			XGBoost	0.831	0.832	0.832	0.664	0.907
Mature RNA	lncRNA	lncRNA	RF	0.766	0.694	0.730	0.461	0.821
			SVM	0.712	0.689	0.700	0.401	0.789
			KNN	0.500	0.500	0.500	0.000	0.734
			LR	0.590	0.802	0.696	0.401	0.797
			XGBoost	0.757	0.703	0.730	0.460	0.784
	lncRNA	mRNA	RF	0.757	0.522	0.639	0.287	0.705
			SVM	0.814	0.424	0.619	0.258	0.717
			KNN	0.493	0.508	0.501	0.002	0.520
			LR	0.804	0.472	0.638	0.292	0.660
			XGBoost	0.652	0.527	0.590	0.181	0.615
	mRNA	lncRNA	RF	0.788	0.608	0.698	0.403	0.807
			SVM	0.761	0.631	0.696	0.395	0.774
			KNN	0.500	0.500	0.500	0.000	0.542
			LR	0.419	0.838	0.628	0.283	0.653
			XGBoost	0.694	0.694	0.694	0.387	0.749
	mRNA	mRNA	RF	0.858	0.825	0.841	0.683	0.916
			SVM	0.840	0.842	0.841	0.682	0.915
			KNN	0.499	0.501	0.500	0.001	0.800
			LR	0.742	0.895	0.819	0.645	0.908
			XGBoost	0.831	0.832	0.832	0.664	0.907

### Construction of an Ensemble Predictor

**Figure 1:**
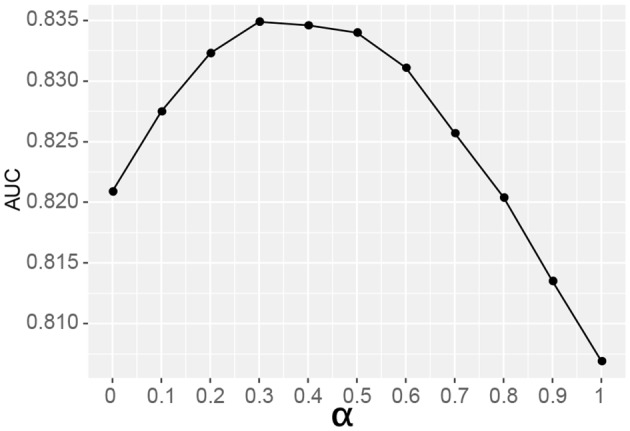
Search for optimal parameter of the ensemble predictor. The optimal result was achieved when α = 0.3. When α = 0, only lncRNA sites were used for training; while when α = 1, only mRNA sites were considered.

Since mRNA methylation sites can also be used for lncRNA site prediction and have achieved a reasonably good performance (Table 3), and considering that we only have a limited number of lncRNA methylation sites, which may not be sufficient for training, an ensemble model using mixed predictive results of mRNA and lncRNA was proposed in order to further improve the lncRNA sites prediction accuracy. The probability of lncRNA sites prediction in this model is defined as follows:

(6) $$\begin{array}{l} P_{en}=\alpha P_{\text{m}}+\left(1-\alpha \right)P_{\text{lnc}} \end{array}$$

where *P*_*en*_ denotes the final prediction probability of the sites in the mature lncRNA mode, *P*_*m*_ represents the prediction probability of the sites when mRNA sites data were used for training, and *P*_lnc_ denotes the prediction probability of the sites when the lncRNA data were used for training. In order to optimize the value of α, which gives the models different weights, a grid search was performed α ∈ [0, 0.1, 0.2, 0.3, 0.4, 0.5, 0.6, 0.7, 0.8, 0.9, 1]. The best performance was achieved when α = 0.3 (AUC = 0.835) (see Figure 1), which indicates that the relatively small number (1,107) of lncRNA sites plays a major role in the ensemble predictor (weight = 0.7), while the very large number (54,476) of mRNA methylation sites plays a minor role (weight = 0.3). The results comparing the mRNA and lncRNA models are shown in Table 4.

**Table 4:** Comparison of ensemble model and lncRNA trained model.

**Predictor**	**Evaluation metrics**				
	**Sn**	**Sp**	**ACC**	**MCC**	**AUC**
mRNA trained	0.788	0.608	0.698	0.403	0.807
lncRNA trained	0.766	0.694	0.730	0.461	0.821
Ensemble (α = 0.3)	0.797	0.689	0.743	0.489	0.835

### Feature Selection

**Figure 2:**
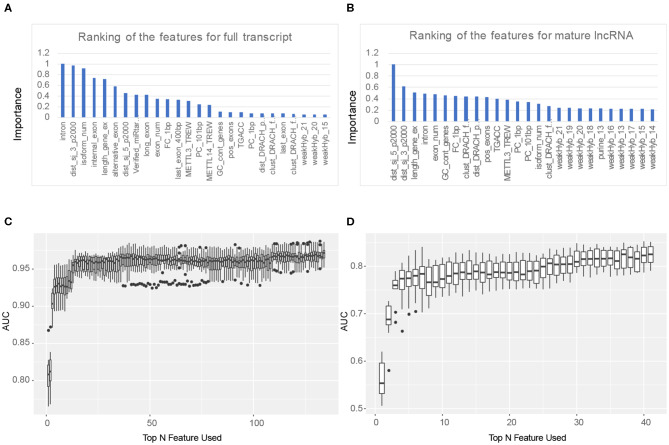
Feature selection results. **(A)** The ranking of the features for full transcript m^6^A site prediction. **(B)** The ranking of the features for mature lncRNA m^6^A site prediction. **(C)** Top 134 features were selected for full transcript m^6^A site prediction. **(D)** Top 41 features were selected for mature lncRNA m^6^A site prediction.

To further optimize the prediction results, we used feature selection to obtain the most effective feature set to predict the methylation sites on lncRNA, and a greedy search was implemented. Firstly, we ranked the features according to their importance through the results of AUC with 10-fold cross validation. Then, one feature was added to the training set each time from the sorted feature set, and the prediction results were obtained using 10-fold cross-validation. The optimal feature set was obtained through the highest AUC. As shown in Figures 2C,D, the first 134 features composed the optimal feature set in the m^6^A sites prediction in the full transcript mode, while the top 41 features can get the highest AUC when predicting m^6^A sites in the mature RNA mode. In addition, it can be seen from Figures 2A,B that the top five features when predicting lncRNA m^6^A sites under the full transcript mode are whether the site is overlapped with the intron (intron), the distance to the downstream (3′ end) splicing junction (dist_sj_3_p2000), the *z*-score of the isoform num (isoform_num), whether the site is overlapped with the internal exon (internal_exon), and the *z*-score of the gene length exons (length_gene_ex). On the other hand, the five most importance features in the prediction sites under the mature RNA mode are the distance to the upstream (5′ end) splicing junction (dist_sj_5_p2000), the distance to the downstream (3′ end) splicing junction (dist_sj_3_p2000), the *z*-score of the gene length exons (length_gene_ex), whether the site is overlapped with the intron (intron), and the *z*-score of the exon num (exon_num). Although some of the first five features are identical in predicting RNA methylation sites in both full transcript and mature lncRNA modes, different characteristics reflect the inherent differences between the two modes.

### Comparison With Existing Methods

**Figure 3:**
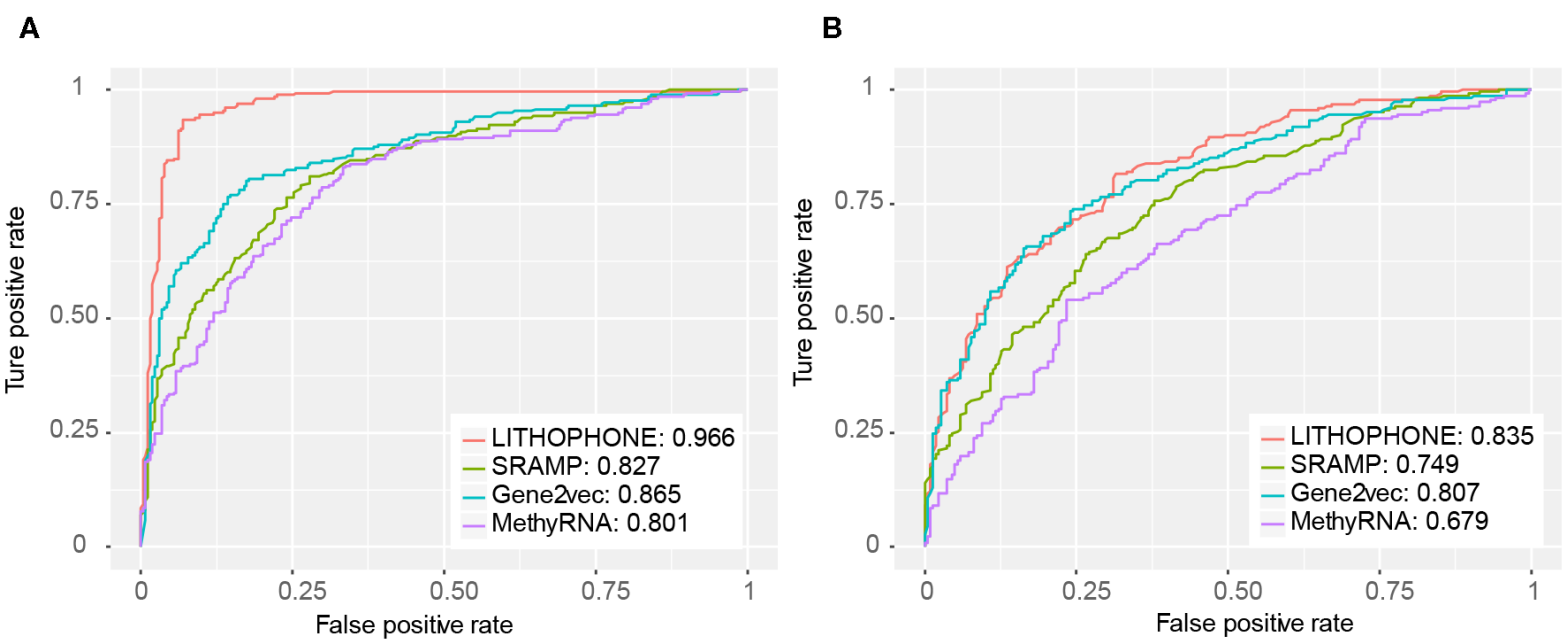
ROC for lncRNA methylation site prediction. The proposed approach substantially outperformed competing approaches. **(A)** The ROC curve for the full transcript mode. **(B)** The ROC curve for the mature RNA mode.

In order to further verify the validity of the proposed algorithm, we compared it with the methods of SRAMP that uses RF to predict mRNA m^6^A sites, MethyRNA that uses the same sequence features as we do, but uses SVM for prediction, and the deep learning method of Gene2vec. These methods have available prediction tools. The results are summarized in Table 5 and the ROC curves of the four methods are shown in Figure 3. The results show that the proposed method is superior to the current popular methods in predicting lncRNA methylation sites.

**Table 5:** Performance comparison for lncRNA m^6^A site prediction.

**Mode**	**Method**	**Evaluation metrics**				
		**Sn**	**Sp**	**ACC**	**MCC**	**AUC**
Full transcript	SRAMP	0.705	0.791	0.748	0.498	0.827
	MethyRNA	0.717	0.752	0.734	0.469	0.801
	Gene2vec	0.798	0.813	0.805	0.611	0.865
	LITHOPHONE	0.922	0.930	0.926	0.853	0.966
Mature RNA	SRAMP	0.604	0.748	0.676	0.355	0.749
	MethyRNA	0.622	0.644	0.633	0.266	0.679
	Gene2vec	0.778	0.689	0.734	0.469	0.806
	LITHOPHONE	0.797	0.689	0.743	0.489	0.835

### LncRNAome-Wide m^6^A Site Prediction

In order to obtain a complete map of all the human lncRNA methylation sites, we searched the entire lncRNAome for all the DRACH motifs, which represent candidate lncRNA methylation sites, under both full transcript and mature RNA modes, and used the proposed method to predict the probability of lncRNA methylation sites. Finally, 330,564 out of the total 4,046,330 DRACH motifs were predicted to contain m^6^A RNA methylation sites under the full transcript mode with a probability greater than 0.5, and 114,093 out of the total 313,458 DRACH motifs from 29,687 lncRNAs were predicted as putative lncRNA methylation sites under the mature RNA mode. The prediction results can be freely accessed at: http://180.208.58.19/lith/. In addition, the data and code used in this article can be obtained from https://github.com/lianliu09/lncRNA-m6a.git.

## Conclusion

With the rapid development of high-throughput sequencing and RNA methylation profiling technologies, people can now study RNA modifications with a high accuracy in the full transcriptome range. In recent years, a number of RNA methylation sites prediction methods have been developed. However, to the best of our knowledge, none of them considered the experimental bias induced in the current epitranscriptome data, which can significantly affect the performance of these predictors.

In this paper, we presented LITHOPHONE, an ensemble framework to predict m^6^A epitranscriptome in lncRNA. Unlike other methods that rely only on sequence information, LITHOPHONE extracts the physicochemical and frequency accumulation characteristics of the bases, combining 60 genomic characteristics to predict the m^6^A methylation modification sites under both full transcript and mature RNA modes on lncRNA using the RF algorithm. To the best of our knowledge, LITHOPHONE is the first m^6^A sites predictor that is optimized for lncRNA. We showed that lncRNA and mRNA exhibit different predictive characteristics, and how LITHOPHONE outperforms competing approaches in lncRNA methylation site prediction. Additionally, we searched the entire lncRNAome in human for all possible m^6^A sites located on lncRNAs and predicted 330,564 m^6^A sites on pre-lncRNA and 114,093 sites on mature lncRNA. We built a website to query the prediction results of lncRNA methylation sites and it is freely accessible at: http://180.208.58.19/lith/. The LITHOPHONE framework can be easily extended to other RNA modifications, such as m^1^A, as well as other species, such as the mouse.

## Data Availability Statement

Publicly available datasets were analyzed in this study. This data can be found here: https://github.com/lianliu09/lncRNA-m6a.git.

## Author Contributions

ZW and LL initialized the project. LL, XL, ZW, and JM designed the research plan. ZW constructed the genomic features considered in site prediction. LL performed the site prediction and drafted the manuscript. ZF and YT built the website. All authors read and critically revised and approved the final manuscript.

## Conflict of Interest

The authors declare that the research was conducted in the absence of any commercial or financial relationships that could be construed as a potential conflict of interest.
