# Supplementary material for: LITHOPHONE: Improving lncRNA Methylation Site Prediction Using an Ensemble Predictor
Source: Front Genet. 2020 Jun 9;11:545. doi: 10.3389/fgene.2020.00545 (PMC7297269; doi:10.3389/fgene.2020.00545)
Supplement: Supplementary file 1 [file Data_Sheet_1.PDF]

Table S1 Genomic features considered in LITHOPHONE

ID	Name	Description	Note
1	TSS	downstream 100bp of transcription start site.	Dummy variables indicating whether the site is overlapped to the topological region on the major RNA transcript.
2	TSS_A	Downstream 100bp of transcription start site with sequence A.	
3	alternative_exon	Alternatively spliced exons.	
4	constitutive_exon	Constitutively spliced exons.	
5	internal_exon	Internal exons.	
6	long_exon	Long exons (length>400bp).	
7	last_exon	The last exon.	
8	last_exon_400bp	5’ start 400 bp of the last exon.	
9	intron	Intron.	
10	pos_exons	relative positioning on exon.	
11	dist_sj_5_p2000	distance to the upstream (5' end) splicing junction.	Nucleotide distances toward the splicing junctions or the nearest neighboring sites.
12	dist_sj_3_p2000	distance to the downstream (3' end) splicing junction.	
13	length_gene_ex	gene length-exons (z-score).	The region length in bp.
14	length_gene_full	gene length full transcript (z-score).	
15	AAACA	motif --- AAACA.	belong to the motif
16	AGACA	motif --- AGACA.	
17	AAACT	motif --- AAACT.	
18	AGACT	motif --- AGACT.	
19	AAACC	motif --- AAACC.	
20	AGACC	motif --- AGACC.	
21	GAACA	motif --- GAACA.	
22	GGACA	motif --- GGACA.	
23	GAACT	motif --- GAACT.	
24	GGACT	motif --- GGACT.	
25	GAACC	motif --- GAACC.	
26	GGACC	motif --- GGACC.	
27	TAACA	motif --- TAACA.	
28	TGACA	motif --- TGACA.	
29	TAACT	motif --- TAACT.	
30	TGACT	motif --- TGACT.	
31	TAACC	motif --- TAACC.	
32	TGACC	motif --- TGACC.	
33	clust_DRACH_f1000	number of DRACH motif neighboors within 1000bp flanking regions.	clustering indicators and motif clustering

34	clust_DRACH_f100	number of DRACH motif neighboors within 100bp flanking regions.	
35	dist_DRACH_p2000	distance to the nearest DRACH motif (peaked at 2000bp).	
36	dist_DRACH_p200	distance to the nearest DRACH motif (peaked at 200bp).	
37	PC_1bp	phast cons scores 1bp.	Scores related to evolutionary conservation
38	PC_101bp	phast cons scores 101bp.	
39	FC_1bp	fitness consequences scores 1bp z score.	
40	FC_101bp	fitness consequences scores 101bp z score.	
41	struct_hybridize	predicted hybridized region.	RNA secondary structures
42	struct_loop	inferred loop structures between hybridized region.	
43	HNRNPC_eCLIP	eCLIP data of HNRNPC RNA binding sites.	RNA annotations related to m^{6}A biology.
44	YTHDC1_TREW	TREW data of YTHDC1 RNA binding sites.	
45	YTHDF1_TREW	TREW data of YTHDF1 RNA binding sites.	
46	YTHDF2_TREW	TREW data of YTHDF2 RNA binding sites.	
47	METTL3_TREW	TREW data of METTL3 RNA binding sites	
48	METTL14_TREW	TREW data of METTL14 RNA binding sites.	
49	WTAP_TREW	TREW data of WTAP RNA binding sites.	
50	METTL16_CLIP	CLIP data of METTL16 RNA binding sites	
51	ALKBH5_PARCLIP	PARCLIP data of ALKBH5 RNA binding sites.	
52	FTO_CLIP	CLIP data of FTO RNA binding sites.	
53	FTO_eCLIP	eCLIP data of FTO RNA binding sites.	
54	TargetScan	Predicted miRNA targeted sites by TargetScan.	
55	Verified_miRtargets	miRNA targeted sites verified by experiment.	
56	miR_targeted_genes	miRNA targeted genes.	a dummy variable indicating wheather

			the lncRNA is a miRNA target
57	isoform_num	isoform number z score.	Isoform and exon number z score
58	exon_num	exon number z score.	
59	GC_cont_genes	gene level GC content z score is generated.	GC content z score
60	GC_cont_101bp	101bp GC content z score.	

Table S2 R packages corresponding to five methods

Method	R package
RF	randomforest
SVM	e1071
KNN	class
LR	e1071
XGBoost	xgboost
